# Morphology and multigene phylogeny of *Talaromycesamyrossmaniae*, a new synnematous species belonging to the section Trachyspermi from India

**DOI:** 10.3897/mycokeys.45.32549

**Published:** 2019-01-28

**Authors:** Kunhiraman C. Rajeshkumar, Neriman Yilmaz, Sayali D. Marathe, Keith A. Seifert

**Affiliations:** 1 National Fungal Culture Collection of India (NFCCI), Biodiversity and Palaeobiology (Fungi) Gr., Agharkar Research Institute, G.G. Agarkar Road, Pune, 411 004, Maharashtra, India National Fungal Culture Collection of India Pune India; 2 Biodiversity (Mycology), Ottawa Research and Development Centre, Agriculture and Agri-Food Canada, 960 Carling Ave., Ottawa, Ontario, K1A 0C6, Canada Agriculture and Agri-Food Canada Ottawa Canada; 3 Department of Microbiology and Plant Pathology, Forestry and Agricultural Biotechnology Institute (FABI), University of Pretoria, Pretoria, South Africa University of Pretoria Pretoria South Africa

**Keywords:** *BenA*, *CaM*, conidial fungi, *RPB2*, synnemata, *Trichocomaceae*, Western Ghats

## Abstract

A new *Talaromyces* species, *T.amyrossmaniae*, isolated from decaying fruit and litter of *Terminalia bellerica*, is described and illustrated. On the natural substrate, the new species produces determinate synnemata, with a well-defined, vivid orange red to orange red cylindrical stipe, and a greyish green capitulum. Conidiophores are typically biverticillate, or sometimes have subterminal branches, with acerose phialides that produce globose to subglobose, smooth to slightly roughened conidia. Multigene phylogenetic analyses based on the internal transcribed spacer region (ITS), and partial sequences of β-tubulin (*BenA*), calmodulin (*CaM*), and DNA directed RNA polymerase second large subunit (*RPB2*) genes, along with morphological characterization, revealed that these isolates are distinct and form a unique lineage of Talaromyces in section Trachyspermi, closely allied to *T.aerius*, *T.albobiverticillius*, *T.heiheensis*, *T.erythromellis*, and *T.solicola*. The new species *T.amyrossmaniae* is the first species in section Trachyspermi with determinate synnemata.

## Introduction

The genus *Talaromyces* was described as a teleomorph-based holomorph genus (Benjamin 1955). It is characterized by cleistothecial ascomata that have a soft hyphal exterior giving them a yellow, cream, pink or reddish coloration; its anamorphs are predominantly biverticillate or rarely terverticillate conidiophores with acerose phialides with a narrow mouth (Samson et al. 2011, Yilmaz et al. 2014). Conventionally, species of *Talaromyces* were linked with *Penicillium*, *Paecilomyces*, *Geosmithia*, and *Merimbla* anamorphs (Pitt 1980, Pitt et al. 2000). Primary phylogenetic studies of *Talaromyces* spp. revealed that they form a distinct clade that includes species formerly classified in PenicilliumsubgenusBiverticillium, separate from *Eupenicillium* and *Penicillium* spp. in other subgenera (LoBuglio et al. 1993; Seifert et al. 1993; Berbee et al. 1995; Peterson 2000; Heredia et al. 2001; Seifert et al. 2004). As redefined following the new single name provision of the International Code of Nomenclature of algae, fungi and plants (ICN), *Talaromyces* was expanded to include asexual species formerly included in PenicilliumsubgenusBiverticillium (Samson et al. 2011; Visagie and Jacobs 2012; Visagie et al. 2012; Yilmaz et al. 2012, 2014). The landmark multigene phylogeny of *Penicillium* and allied genera by Houbraken and Samson (2011) segregated the prevailing concept of the family Trichocomaceae into three families, Aspergillaceae, Thermoascaceae, and Trichocomaceae. *Talaromyces* sensu stricto is presently classified in the Trichocomaceae along with *Thermomyces*, *Sagenomella*, *Rasamsonia*, and *Trichocoma*. The molecular taxonomy and nomenclature *Talaromyces* were comprehensively revised in the recent past (Houbraken and Samson 2011; Samson et al. 2011; Seifert et al. 2012; Visagie and Jacobs 2012; Visagie et al. 2012; Yilmaz et al. 2012; Yilmaz et al. 2014). Yilmaz et al. (2014) resolved the phylogenetic positioning of *Talaromyces* species using a polyphasic taxonomic concept and placing 88 accepted species in seven well-defined sections, namely *Bacillispori*, *Helici*, *Islandici*, *Purpurei*, *Subinflati*, *Talaromyces*, and *Trachyspermi*. Subsequent to the monograph by Yilmaz et al. (2014), 54 new *Talaromyces* species have been described from all over the world (Visagie et al. 2015; Chen et al. 2016; Luo et al. 2016; Crous et al. 2016; Romero et al. 2016; Wang et al. 2016; Wang et al. 2016; Yilmaz et al. 2016a, b; Crous et al. 2017; Guevara-Suarez et al. 2017; Peterson and Jurjević 2017; Wang et al. 2017; Barbosa et al. 2018; Su and Niu 2018; Jiang et al. 2018; Varriale et al. 2018).

During the 2009 monsoon season, routine surveys were conducted to explore microfungal diversity in natural forests of Lingmala waterfalls area (17.9218N; 73.6870E) of Mahabaleshwar, northern Western Ghats, India. A previously undescribed synnema-forming fungus with penicillate conidiophores and phialidic conidiogenous cells was collected from decaying fruits and litter of *Terminalia bellerica* (*Combretaceae*) fallen onto the ground near the Lingmala waterfalls. The fungus was isolated into pure culture on different culture media, microscopic characters were recorded and its classification studied using phylogenetic analysis of aligned DNA sequences from the nuclear ribosomal ITS region and *BenA*, *CaM*, and *RPB2* partial gene sequences. This paper aims to resolve the taxonomy and phylogeny of this synnematous species, which is shown to represent a new species in TalaromycessectionTrachyspermi, here named *T.amyrossmaniae*.

## Materials and methods

### Isolation

Conidia were removed from synnemata directly from the surface of fallen fruits under a Nikon stereomicroscope (model SMZ1500 with Digital camera; Nikon, Tokyo, Japan) and placed on malt extract agar (MEA) media containing the antibiotic Streptomycin sulphate (100 mg/L) CMS220-5G (HIMEDIA Laboratories Pvt. Ltd, Mumbai, India). Methods and media used for examining colony characters, inoculating and incubating cultures, and microscopic examination followed those of Visagie et al. (2014), with the addition of Oatmeal Agar (OA), and Potato Dextrose Agar (PDA), with incubation occurring in a Bio Multi Incubator (Model LH-30-8CT, Japan). Herbarium specimens were deposited in the Ajrekar Mycological Herbarium (AMH); cultures were accessioned and preserved in the National Fungal Culture Collection of India (NFCCI; WDCM-932), Agharkar Research Institute, Pune, India. Reference and ex-type strains used in this study are listed in Table 1.

**Table 1.** Accession numbers for fungal strains and strains used for the phylogenetic analysis.

Species	Collection no.	Substrate and origin	GenBank accession no.			
			ITS	*BenA*	*CaM*	*RPB2*
*T.aerius*	CBS 140611^T^	Indoor air, China	KU866647	KU866835	KU866731	KU866991
*T.albobiverticillius*	CBS 133440^T^	Decaying leaves of a broad leaved tree, Taiwan	HQ605705	KF114778	KJ885258	KM023310
	CBS 140498	Air from HVAC system, China	KR855658	KR855648	KR855653	KR855663
***T.amyrossmaniae***	**NFCCI 1919^T^**	**Fallen decaying fruits of *Terminalia bellerica* (Combretaceae), Maharashtra, India**	**MH909062**	**MH909064**	**MH909068**	**MH909066**
	**NFCCI 2351**	**Fallen decaying fruits of *Terminalia bellerica* (Combretaceae), Maharashtra, India**	**MH909063**	**MH909065**	**MH909069**	**MH909067**
*T.assiutensis*	CBS 147.78^T^	Soil, Egypt	JN899323	KJ865720	KJ885260	KM023305
	CBS 645.80	*Gossypium*, India	JN899334	KF114802	*	*
*T.atroroseus*	CBS 133442^T^	House dust, South Africa	KF114747	KF114789	KJ775418	KM023288
	CBS 133449	Mouse dung, Denmark	KF114744	KF114788	*	*
*T.austrocalifornicus*	CBS 644.95^T^	Soil, USA	JN899357	KJ865732	KJ885261	*
*T.brasiliensis*	CBS 142493^T^	Honey of *Meliponascutellaris*; Recife, Pernambuco, Brazil	MF278323	LT855560	LT855563	LT855566
*T.convolutus*	CBS 100537^T^	Soil, Nepal	JN899330	KF114773	*	JN121414
*T.diversus*	CBS 320.48^T^	Leather, USA	KJ865740	KJ865723	KJ885268	KM023285
	DTO 244-E6	House dust, New Zealand	KJ775712	KJ775205	*	*
*T.erythromellis*	CBS 644.80^T^	Soil from creek bank, New South Wales	JN899383	HQ156945	KJ885270	KM023290
*T.heiheensis*	HMAS 248789^T^	Rotten wood, China	KX447526	KX447525	KX447532	KX447529
*T.minioluteus*	CBS 642.68^T^	Unknown	JN899346	KF114799	KJ885273	JF417443
	CBS 270.35	*Zeamays*, USA	KM066172	KM066129	*	*
	CBS 137.84	Fruit damaged by insect, Spain	KM066171	KF114798	*	*
*T.minnesotensis*	CBS 142381^T^	Human ear, USA	LT558966	LT559083	LT795604	LT795605
*T.solicola*	DAOM 241015^T^	Soil, South Africa	FJ160264	GU385731	KJ885279	KM023295
	CBS 133446	Soil, South Africa	KF114730	KF114775	*	*
*T.systylus*	BAFCcult3419^T^	Soil, Argentina	KP026917	KR233838	KR233837	*
*T.trachyspermus*	CBS 373.48^T^	Unknown, USA	JN899354	KF114803	KJ885281	JF417432
	CBS 118437	Soil, Morocco	KM066169	KM066127	*	*
*T.ucrainicus*	CBS 162.67^T^	Unknown	JN899394	KF114771	KJ885282	KM023289
	CBS 127.64	Soil treated with cyanimide, Germany (ex-type of *T.ohiensis*)	KM066173	KF114772	*	*
*T.udagawae*	CBS 579.72^T^	Soil, Japan	JN899350	KF114796	KX961260	*

^T^: ex-type strain

### Morphology

Colony characters were recorded after 7 d of incubation on various media, including Czapek yeast autolysate agar (CYA), Blakeslee’s (1915) malt extract agar (MEAbl), yeast extract sucrose agar (YES), oatmeal agar (OA), and creatine sucrose agar (CREA). Bacto malt extract was used for MEAbl. Media preparation, inoculations, incubation conditions, and microscopic preparations followed the recommendations by Visagie et al. (2014). Colour codes and names used in descriptions are from Kornerup and Wanscher (1967). Microscopic observations were made with an Olympus (Model CX-41, Japan) dissecting microscope and Zeiss (AXIO Imager 2, Germany) compound microscope equipped with Nikon Digital sight DS-Fi1 and AxioCam MRc5 cameras driven by AxioVision Rel 4.8 software (AXIO Imager 2, Germany).

### DNA extraction, amplification, and phylogenetic analyses

Colonies were grown on MEAbl plates, and genomic DNA was extracted following the rapid salt extraction method of Aljanabi and Martinez (1997). The ITS regions was amplified using primer pairs ITS5 and ITS4 (White et al. 1990). For the amplification of *RPB2* gene region, primer pairs RPB2-5F and RPB2-7cR (Liu et al. 1999) were used with touch-up PCR conditions: 5 cycles with annealing temperature 48 °C followed by 5 cycles at 50 °C and final 25 cycles at 52 °C. The partial *BenA* gene was amplified with primer pair Bt2a and Bt2b (Glass and Donaldson 1995) with 50 °C as annealing temperature. The partial *CaM* gene was amplified using primer pair CF1M and CF4 (Hubka et al. 2014) subjected to 32 cycles under the following temperature regime: first cycle at 95 °C for 3 min, 55 °C for 30 seconds, and 72 °C for 1 min; followed by 30 cycles at 95 °C for 30 seconds, 55 °C for 30 seconds, 72 °C for 1 min; and a final cycle at 95 °C for 30 seconds, 55 °C for 30 seconds, and 72 °C for 10 min. PCR products were purified with StrataPrep PCR Purification Kit (Agilent Technologies, TX, USA) and sequenced using the BigDye Terminator v. 3.1 Cycle Sequencing Kit (Applied Biosystems, USA). Sequencing reactions were run on a ABI PRISM® 3100 Genetic Analyzer (Applied Biosystems, USA).

### Sequence alignment and phylogenetic analysis

Reference sequences of TalaromycessectionTrachyspermi were downloaded from GenBank and aligned in MAFFT v. 7.305b (Katoh and Standley 2013) with the newly generated sequences. Alignments were manually adjusted in Geneious as needed. A Maximum Likelihood analysis was done in IQtree v. 1.6 (Nguyen et al. 2015) after selecting the most suitable substitution model with the Modelfinder (Kalyaanamoorthy) algorithm built into the software. The trees were visualized in Figtree v. 1.4.3 ((http://tree.bio.ed.ac.uk/software/figtree) and edited for publication in Affinity Designer v. 1.6.1 (Serif Europe Ltd, UK). The new DNA sequences were deposited in GenBank (Table 1).

## Results

### Phylogenetic analyses

**Figure 1.**
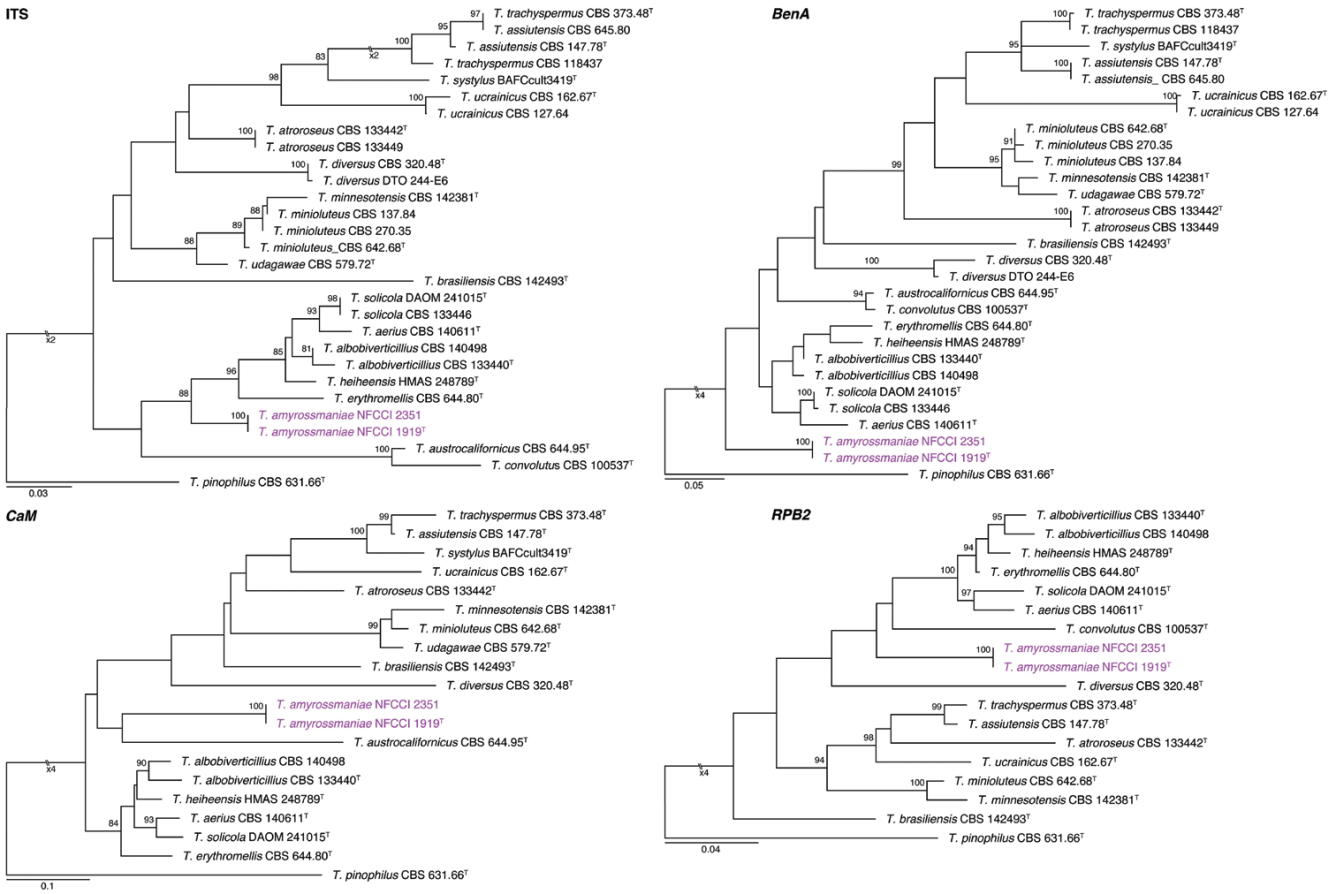
Maximum likelihood (ITS) phylogenetic trees of ITS region, *BenA, CaM* and *RPB2* genes of strains belong TalaromycessectionTrachyspermi. *Talaromycespinophilus* (CBS 631.66^T^) was chosen as outgroup. Bootstrap values above 70% are indicated. Purple names indicate *T.amyrossmaniae* strains. ^T^: ex-type.

**Figure 2.**
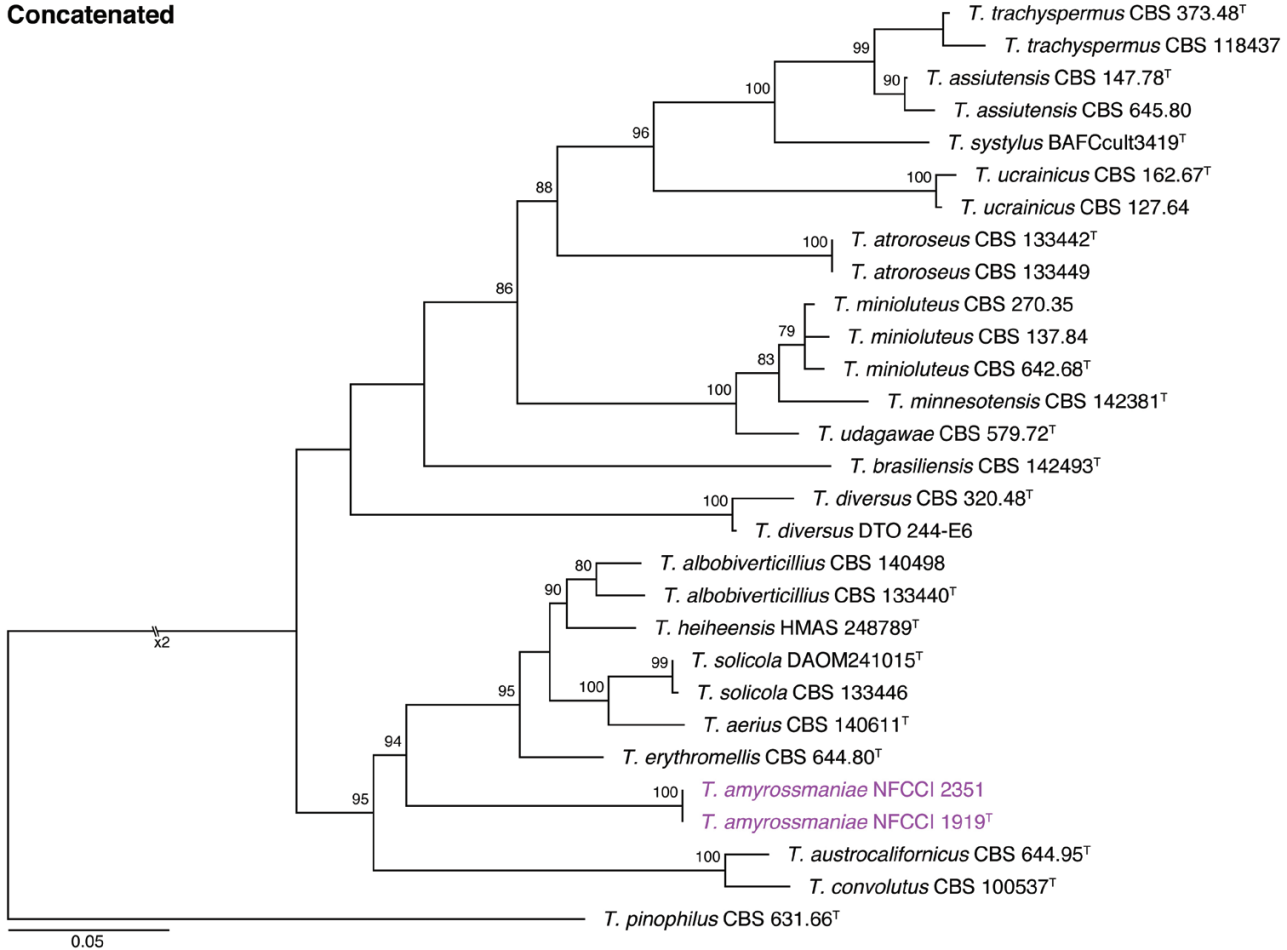
Maximum likelihood (ITS) combined phylogenetic trees using ITS region, *BenA, CaM* and *RPB2* genes of strains belong TalaromycessectionTrachyspermi. *Talaromycespinophilus* (CBS 631.66^T^) was chosen as outgroup. Bootstrap values above 70% are indicated. Purple names indicate *T.amyrossmaniae* strains. ^T^: ex-type.

The phylogenetic analysis showed that the new species described below as *Talaromycesamyrossmaniae* belongs to section Trachyspermi. The relationships of the new species with accepted species and its genetic coherence and phylogenetic consistency were analysed with single concatenated sequence datasets based on four loci (ITS, *BenA, CaM* and *RPB2*). The length of the data sets were 540 bp for ITS, 379 bp for *BenA*, 550 bp for *CaM*, and 851 bp for *RPB2* loci. The best fitting models for the ITS analysis were TPM2u+F+I+G4 for ITS, TIM2e+G4 for *BenA*, K2P+I+G4 for *CaM*, and K2P+I+G4 for *RPB2*. All trees were rooted with *T.pinophilus* (CBS 631.66). The single gene trees and the multigene phylogram are shown in Figures 1, 2.

Because of the limited resolution of the official fungal DNA barcode, the ITS (Schoch et al. 2012), in the *Trichocomaceae*, *BenA* was proposed as the secondary DNA barcode for *Talaromyces* (Yilmaz et al. 2014). The overall tree topologies of ITS and *BenA* phylogenies had relatively consistent association of species. However, the type species of section Trachyspermi, *T.trachyspermus* was well separated from *T.assiutensis* in the *BenA* analysis whereas strains of the two species were intermixed in the ITS analysis. *Talaromycesucraicinus* was consistently a sister clade to *T.trachyspermus* and *T.assiutensis*. Our proposed new species, *T.amyrossmaniae*, was distinguished from other species both by ITS and other markers (Figs 1, 2). It is consistently included in a major clade along with *T.aerius*, *T.albobiverticillius*, *T.erythromellis*, *T.heiheensis*, and *T.solicola* in the ITS analysis. As with the ITS, in the concatenated phylogeny and *RPB2* analyses, *T.amyrossmaniae* clustered with *T.albobiverticillius*, *T.heiheensis*, *T.erythromellis*, *T.aerius*, and *T.solicola* (Figs 1, 2). However, in the *BenA* phylogeny, *T.amyrossmaniae* was segregated from that major-clade. *Talaromycesamyrossmaniae* is clustered with *T.austrocalifornicus* in the *CaM* analyses (Fig. 1). Yilmaz et al. (2014) mentioned that amplification of *CaM* is difficult in section Trachyspermi. *CaM* data could not be analyzed critically because the new sequences generated through Sanger sequencing (ABI PRISM 3100 Genetic Analyzer) contained a homopolymer stretch of around 30 bp after base 320, resulting in poor quality and short sequences, even after many attempts using modified PCR conditions and primers.

### Morphology

In this study, we introduce one new species, *Talaromycesamyrossmaniae* belonging to section Trachyspermi. Strains conform with the general morphological characters of this section. *Talaromycesamyrossmaniae* was compared with its close relatives, with the distinguishing characters mentioned in the note after the species description. Also, Table 2 compares the new species with the closely allied species in section Trachyspermi. The main character that differentiates *T.amyrossmaniae* from other synnemata-producing species in the genus *Talaromyces* is the length of the synnemata. *Talaromycesamyrossmaniae* has the shortest synnemata (up to 150 µm). A synopsis of comparative morphology and growth rate of synnema producing species of *Talaromyces* is given in Table 3.

**Table 2.** Comparative morphology of TalaromycessectionTrachyspermi.

Species	Conidiophore branching	Conidia ornamentation	Conidial shape	Conidial size (µm)
*T.aerius*	Biverticillate, minor proportion with subterminal branches	Smooth	Ellipsoidal	2–3.5 (–4.5) × 2–3
*T.albobiverticillius*	Biverticillate, minor proportion with subterminal branches	Smooth to finely roughened	Globose to subglobose	2–3.5 (–4) × 1.5–2.5
***T.amyrossmaniae***	**Biverticillate, minor proportion with subterminal branches**	**Smooth to finely roughened**	**Globose or subglobose**	**2.5–4 (–6) × 2.5–3.5 (–8)**
*T.assiutensis*	Mono to biverticillate	Smooth	Ovoidal to ellipsoidal	2–4 × 1.5–2.5
*T.atroroseus*	Biverticillate, minor proportion with subterminal branches	Finely roughened to rough	Ellipsoidal	2–3.5 × 1.5–2.5
*T.austrocalifornicus*	Biverticillate	Smooth	Subglobose	1.5–3 × 1.5–2.5
*T.brasiliensis*	Biverticillate	Finely roughened	Globose	2 × 3
*T.convolutus*	Mono to biverticillate	Smooth	Ellipsoidal	(2–) 3–4 × 1.5–2 (–3)
*T.diversus*	Biverticillate, minor proportion with subterminal branches	Smooth to finely roughened	Subglobose to ellipsoidal	2–3 (–5) × 2–3 (–3.5)
*T.erythromellis*	Biverticillate having symmetrical subterminal branches,	Smooth	Subglobose to ellipsoidal	2–3.5 × 1.5–2.5
*T.heiheensis*	Biverticillate with subterminal branches, minor proportionquaterverticillate	Smooth	Subglobose to ellipsoidal	2.5–3 × 2–2.5
*T.minioluteus*	Biverticillate	Smooth	Ellipsoidal	2.5–4 × 1.5–2.5
*T.minnesotensis*	Biverticillate	Smooth	Ellipsoidal	2.5–3.5 × 2–3
*T.solicola*	Biverticillate	Rough	Globose to subglobose	2–3.5 × 2–2.5
*T.systylus*	Biverticillate	Rough	Globose	3.5 × 4
*T.trachyspermus*	Mono to biverticillate	Smooth	Ellipsoidal	2–3.5 (–5) × 1.5–2.5
*T.ucrainicus*	Mono to biverticillate	Smooth	Broadly ellipsoidal to ovoidal	2–4 (–5) × 1.5–2.5 (–3)
*T.udagawae*	Biverticillate	Smooth	Subglobose to ellipsoidal	3–4 × 2–3

**Table 3.** Synopsis of comparative morphology and growth rate of synnema producing species of *Talaromyces*.

Species	Section	Synnemata			Growth rates (mm)			
		Shape	Time of production	Height (µm)	Acid production on CREA	CYA 25 °C	CYA 37 °C	MEA 25 °C
*T.amyrossmaniae* ^a^	*Trachyspermi*	Determinate	Prolonged	Up to 150	Absent	4–6	No growth	12–14
*T.calidicanius* ^b^	*Talaromyces*	Determinate	Prolonged	Up to 6000	Moderate	27–30	No growth	47–48
*T.cecidicola* ^b^	*Purpurei*	Determinate	Prolonged	Up to 1250	Absent	33–34	No growth	37–38
*T.choloroloma* ^b^	*Purpurei*	Determinate	Prolonged	Up to 1200	Weak to moderate	40–45	No growth	45–48
*T.coalescens* ^b^	*Purpurei*	Determinate	Prolonged	Up to 1200	Very weak	32–34	2–4	43–45
*T.dendriticus* ^b^	*Purpurei*	Determinate	Prolonged	Up to 5000	Absent	23–26	5–6	35–36
*T.duclauxii* ^b^	*Talaromyces*	Indeterminate	After 7d	Up to 5000	Weak	25–27	3–4	48–50
*T.flavovirens* ^b^	*Talaromyces*	Determinate, covered or masked by yellow mycelial covering	Prolonged	Up to 750	Absent	19–20	5–6	37–38
*T.palmae* ^b^	*Subinflati*	Indeterminate	Prolonged	Up to 8000	Weak	20–25	No growth	22–26
*T.panamensis* ^b^	*Talaromyces*	Determinate, cone shaped and often sterile	After 7d	Up to 6800	Strong	23–24	No growth	28–30
*T.pittii* ^b^	*Purpurei*	Determinate, phototropic	Prolonged	Up to 1000	Absent	34–36	No growth	42–44
*T.pseudostromaticus* ^b^	*Purpurei*	Determinate	Prolonged	Up to 8000	Absent	25–34	No growth	38–43
*T.ramulosus* ^b^	*Purpurei*	Determinate	Prolonged	Up to 500	Absent	32–40	5–8	45–48
*T.systylus* ^c^	*Trachyspermi*	Indeterminate	Prolonged	Up to 4000	Good	14–18	16–19	18–21

^a^: Data from this study. ^b^: Data from Yilmaz et al. (2014). ^c^: Data from Romero et al. (2016).

## Taxonomy

### Talaromyces amyrossmaniae

Taxon classificationFungiEurotialesTrichocomaceae

Rajeshkumar, Yilmaz & Seifert sp. nov.

518601

Figure 3

#### Etymology.

Latin, named after Dr Amy Y. Rossman, in honour of her career as a research leader in Systematic Mycology and Microbiology, USDA ARS, Beltsville, Maryland, USA.

#### Diagnosis.

Synnemata abundant in nature, determinate, 90–120 µm tall, with an unbranched stalk 10–35 µm wide, base wider, up to 50–60 µm. Synnema stipe orange red or vivid orange red, capitulum terminal, compact, globose with conidiophores and a powdery grey-green conidial mass in closely packed, split columns. On MEAbl synnemata produced after 2 weeks incubation, up to 120 µm long. Conidiophores biverticillate, with sometime terverticilate sub-branches. Acerose phialides producing smooth to slightly roughened globose to subglobose conidia. Restricted growth on all media, acid production absent on CREA.

In: TalaromycessectionTrachyspermi.

#### Type.

INDIA, Maharashtra, Mahabaleshwar, Lingmala falls; isolated from fallen decaying fruits and litter of *Terminalia bellerica* (*Combretaceae*), 9 June 2009, isolated by K.C.Rajeshkumar, holotype: AMH 9330, extype: NFCCI 1919, other culture NFCCI 2351.

Gene sequences: ex-holotype MH909062(ITS), MH909064(*BenA*), MH909068(*CaM*), MH909066(*RPB2*).

#### Description.

Colony diameter, 7 d (mm): CYA 4–6; CYA 37 °C no growth; MEAbl 12–14; YES 5–7; DG18 4–5; OA 10–13; CREA 3–5.

Colony characters: CYA 25 °C, 7 d: colonies low, plane; margins low, entire (< 1 mm); mycelia white; no germination; sporulation absent; soluble pigmentation absent; exudates absent; reverse Yellowish white (4A2). MEAbl 25 °C, 7 d: colonies low, slightly raised, synnemata present; margins low, entire (1 mm); mycelia white; texture velvety; sporulation dense (except margins); conidia en masse Dull green (27D4–27E4); soluble pigmentation yellow; exudates orange to reddish orange small droplets; reverse Hazel brown (6E6) at center fading into Light brown (6D8) to Light yellow (3A5). YES 25 °C, 7 d: colonies slightly raised, sulcate, sunken at center; margins low, entire (< 1 mm); mycelia pale pinkish red, with and appearance of Pastel red (7A4–7A5); texture floccose; sporulation absent; soluble pigmentation absent; exudates absent; reverse Light brown (7D6). DG18 25 °C, 7 d: colonies slightly raised, sulcate, sunken at center; margins low, entire (1 mm); mycelia pale yellow; texture floccose; sporulation moderately dense at center, margins absent; conidia en masse Greyish green to Dull green (26C4–26D4); soluble pigmentation absent; exudates absent; reverse Brownish orange to Brownish yellow (5C6) in the center, fading into Light yellow (4A5). OA 25 °C, 7 d: colonies low, plane; margins low, entire (< 1 mm); mycelia white; texture velvety; sporulation moderately dense at center, margins absent; conidia en masse Greyish green (27C4–27D4); soluble pigmentation dark red; exudates absent; reverse Brown (7E8) in the centre, fading into Copper red (7C8). CREA 25 °C, 7 d: acid production absent.

**Figure 3.**
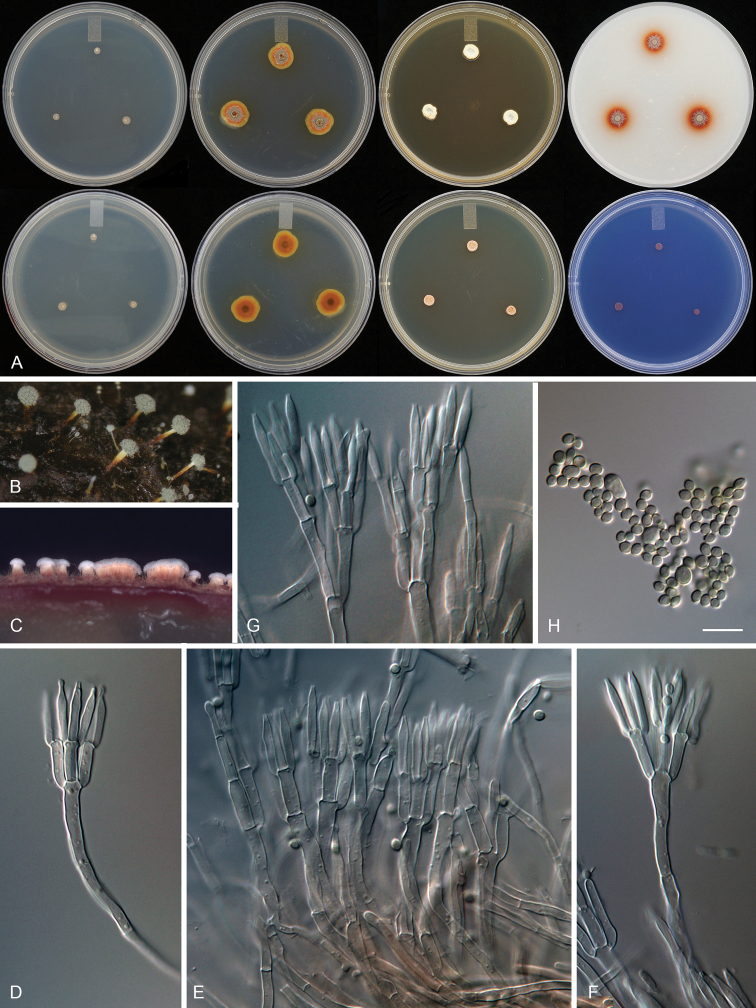
*Talaromycesamyrossmaniae* (NFCCI 1919) **A** Colonies on CYA, MEAbl (obverse and reverse), Colonies obverse on YES, OA, DG18, CREA**B** Synnemata on *Terminalia bellerica* fruit in nature **C** Synnema formation on MEAbl after 14 d at 25 °C **D–F** Biverticillate penicilli **G** Biverticillate penicilli with subterminal branches **H** Conidia. Scale bar: 10 µm.

#### Micromorphology.

Determinate synnemata formed after 2 weeks on MEAbl up to 80–150 µm long. Conidiophores biverticillate with a minor proportion having subterminal branches; stipes smooth walled 80–120 × 3–4 µm; extra branches up to 30 µm long; metulae three to six, divergent, 10–13 × 2.5–3 µm; phialides acerose, three to six per metulae, 12–15 (–18) × 2–3 µm; conidia smooth, globose to subglobose, 2.5–4 × 2.5–3.5 µm. Sometimes it produces large-sized conidia up to 6–8 µm. Ascomata not observed.

## Discussion

In our study, a novel *Talaromyces* species, *T.amyrossmaniae* is described based on two isolates from decaying fruits and litter of *Terminalia bellerica* (*Combretaceae*). We used ITS, *BenA*, *CaM* and *RPB2* sequences to apply genealogical concordance phylogenetic species recognition (GCPSR; Taylor et al. 2000) to delineate the species, and a multigene phylogenetic analysis to place *T.amyrossmaniae* in TalaromycessectionTrachyspermi. TalaromycessectionTrachyspermi (as ‘*trachyspermus*’) was introduced by Yaguchi et al. (1996) based on ubiquinone systems, overriding the traditional morphology based classification of *Talaromyces*. Yilmaz et al. (2014) applied multigene phylogenies and morphology to redefine classification of *Talaromyces* and divided the genus into seven sections. They noted that TalaromycessectionTrachyspermi includes species with generally biverticillate penicilli with acerose phialides and when ascomata are produced, they are creamish white or yellow. Colonies generally grow restrictedly on CYA, YES, CREA and DG18; some species have colonies with abundant red pigments.

Morphologically, *T.amyrossmaniae* resembles the other species of section Trachyspermi and produces dark orange to red pigmentation on MEAbl, restricted growth on MEAbl, CYA, DG18, YES and CREA, symmetrical biverticillate penicilli with a minor proportion having sub-terminal branches, and acerose phialides that form globose to subglobose, smooth to slightly roughened conidia. Although, synnematous Talaromyces species also are found in section Purpurei, section Talaromyces, section Trachyspermi, and section Subinflati, *T.amyrossmaniae* is the first species in section Trachyspermi with determinate synnemata that are seen on fallen decaying fruits in nature and also on MEAbl after 7–14 d of incubation at 25 °C. *Talaromycessystylus* is another synnema producer in section Trachyspermi, but it produces indeterminate synnemata up to 4000 µm and grows at 37 °C (Romero et al. 2016). *Talaromycesamyrossmaniae* has the shortest synnemata in *Talaromyces* and it grows very restrictedly compared to the other synnema producing species on CYA and MEAbl. With these key characters, it is easy to distinguish the new species from the other synnemata producing species of *Talaromyces*.

Based on ITS, *BenA, CaM*, and *RPB2* phylogenies, *T.amyrossmaniae* is part of the same clade as *T.austrocalifornicus*, *T.convolutus*, *T.heiheensis*, *T.aerius*, *T.solicola*, *T.albobiverticillius*, and *T.erythromellis*; however, it can be distinguished from all of these species by having determinate synnemata in nature and by differences in colony growth characteristics. Also, *T.amyrossmaniae* forms predominant concentric rings of synnemata on the different media used in our studies and even forms synnemata in vitro on MEAbl.

Many species of *Talaromyces* have been recorded as saprophytes, endophytes, and human pathogens from different geoclimatic regions and microhabitats across India. Most importantly, *Talaromycesmarneffei* is a potentially pathogenic thermally dimorphic fungus causing systemic mycosis in HIV-infected patients; its dissemination was thoroughly studied from Manipur state of India (Singh et al. 1999; Ranjana et al. 2002). Recent studies on endophytic *T.pinophilus* isolated from the rhizomes of *Curcumaamada* from Karnataka revealed the production and partial characterization of L-asparaginase (Krishnapura and Belur 2016). Likewise, endophytic *T.radicus*, isolated from *Catharanthusroseus*, produces vincristine and vinblastine and was studied for induce apoptotic cell death (Palem et al. 2015). *Talaromycesflavus* is also recorded as an endophytic fungi isolated from ethno-medicinal plants in the sacred forests of Meghalaya having antimicrobial and antioxidant activity (Bhagobaty and Joshi 2012). Devi et al. (2014) reported a marine strain of *T.verruculosus*, from Andhra Pradesh, as a potent polyhydroxybutyrate degrader. Similarly, the stress-tolerant soil fungus *T.funiculosus*, isolated from the neem rhizosphere, was identified as a potential strain for phosphate solubilization (Kanseet al. 2015). *Talaromycesflavus* isolated from paddy rhizosphere of Darjeeling Hills exhibited phosphate solubilizing activity in vitro and positively influenced the growth of *Oryzasativa*, *Cicerarietinum*, and *Vignaradiata* under greenhouse conditions (Chakraborty et al. 2011). A keratin degrading strain of *T.trachyspermus* was isolated from the grounds of a gelatin factory in Jabalpur, Madhya Pradesh, and digested human hair in stationary culture (Rajak et al. 1991). *Talaromycestrachyspermus* was reported as a soil saprophyte in paddy fields of Orissa (Dutta and Ghosh 1965). Species identifications of these *Talaromyces* strains were mostly based on micro- and macro morphological characters in these studies. Because such approaches often underestimate species diversity, adoption of a polyphasic approach to authenticate such identifications will increase the number of *Talaromyces* species known from different eco-geographic zones of India. Further investigation is also needed to study the ecological importance of these species.

## Supplementary Material

XML Treatment for Talaromyces amyrossmaniae
